# Evaluation of *in vivo* antitrypanosomal activity of crude extracts of *Artemisia abyssinica* against a*Trypanosoma congolense* isolate

**DOI:** 10.1186/1472-6882-14-117

**Published:** 2014-04-01

**Authors:** Teka Feyera, Getachew Terefe, Workineh Shibeshi

**Affiliations:** 1 Department of Biomedical Sciences, College of Veterinary Medicine, Jigjiga University, Jigjiga, Ethiopia; 2 Department of Pathology and Parasitology, College of Veterinary Medicine and Agriculture, Addis Ababa University, P.O. Box34, Debrezeit, Ethiopia; 3 Department of Pharmacology and Clinical Pharmacy, School of Pharmacy, College of Health Sciences, Addis Ababa University, P.O. Box 9086, Addis Ababa, Ethiopia

**Keywords:** *Artemisia abyssinica*, Antitrypanosomal activity, *Trypanosoma congolense*, Mice

## Abstract

**Background:**

African trypanosomiasis is a major disease of economic and public health importance affecting agricultural and human development. The search for alternative compounds against African trypanosomiasis is justified by various limitations of existing chemotherapeutic agents. This study was aimed at screening the hydromethanolic and dichloromethane (DCM) crude extracts of aerial parts of *Artemisia abyssinica* for *in vivo* antitrypanosomal activity against *Trypanosoma congolense* isolate in mice.

**Methods:**

The aerial parts of the plant were extracted by maceration technique using dichloromethane and 80% methanol to obtain the corresponding crude extracts. The plant extracts at doses of 100, 200 and 400 mg/kg body weight were administered intraperitoneally daily for 7 days to mice infected with *Trypanosoma congolense*. Diminazene aceturate and distilled water were used as positive and as negative controls respectively. The level of parasitaemia, body weight, packed cell volume, differential leukocyte counts and mean survival period were monitored.

**Results:**

The study showed that the DCM extract at 200 and 400 mg/kg, and the hydromethanolic extract at 400 mg/kg reduced parasitaemia (p < 0.05), ameliorated anaemia (p < 0.05), prevented body weight loss (p < 0.05) and resulted in significant increase in neutrophil levels (p < 0.05) and marked decrease in lymphocyte levels (p < 0.05) compared to the negative control.

**Conclusions:**

This study established that aerial parts of *A. abyssinica* have antitrypanosomal potential and can be considered a potential source of new drugs for the treatment of tropical diseases caused by trypanosomes.

## Background

African trypanosomiasis, a parasitic infection caused by single-celled protozoan parasites of the genus *Trypanosoma* that are primarily transmitted by the bite of infected tsetse flies. Human trypanosomiasis is caused by two sub-species of *Trypanosoma brucei*: *T. brucei gambiense*, and *T. brucei rhodesiense* while African animal trypanosomiasis is a group of diseases of ruminants, camels, equines, swine and carnivores caused by different trypanosome species [1-3]. Both, human and animal trypanosomiases negatively affect the whole economy of Africa by weakening both human and animals health [4].The unavailability of vaccine against trypanosomiasis and escalating costs of initiating and maintaining tsetse control campaigns have led to the vast tsetse infested areas of Africa being almost completely reliant on the use of trypanocidal drugs [5]. However, only a small group of chemoprophylactic and chemotherapeutic trypanocidal compounds are currently in use to which resistance has developed [6]. Furthermore, the registered trypanocides are beset with several additional drawbacks such as frequent toxicity, lengthy parenteral administration, lack of efficacy and unaffordable price [7]. The need for alternative new molecules that are safe, effective and affordable is urgent. It has been observed that natural products derived from plants offer novel possibilities to obtain new drugs that are active against trypanosomes and investigation of antitrypanosomal activity of traditionally used plants has been a major area of contemporary research focus [8]. In recent years, very small Ethiopian potential medicinal plants have been studied for their antitrypanosomal activity [9,10]. There is a need for the development of new agents to complement the existing drugs for the treatment of African trypanosomiasis. *Artemisia abyssinica,* known by various common names in Ethiopia, is used as a traditional remedy for several infectious and noninfectious diseases including typanosomiasis [11-13]. Nibret and Wink [9] reported the *in vitro* antitrypanosomal activity and cytotoxicity of the plant against drug sensitive *T. brucei*. Our former report [14] shows significant *in vitro* antitrypanosomal activity of crude extracts of aerial parts of the plant against *T. congolense.* Therefore, this study was aimed to evaluate the *in vivo* antitrypanosomal activity of crude extracts of aerial parts of the plant against field isolate of *T. congolense* in mice models of infection.

## Methods

### Chemicals

The chemicals and reagents used for the experiment include absolute methanol (Cheshire, UK), dichloromethane (ReAgent Chemical Services Ltd, UK), Tween-80 (BDH Laboratory supplies Ltd, England), phosphate buffered saline (PBS), 10% Giemsa stain (Shenyang Xin Guang, China), diminazene aceturate (Techno Pharmchem, India).

### Plant material

Fresh aerial parts of *A. abyssinica* were collected in November 2011 from Sebeta, Oromia Regional State, about 24 km South of Addis Ababa, Ethiopia. The fresh plant material was wrapped with plastic sheets during transportation. The plant specimen was identified by a taxonomist in the Department of Biology and voucher specimen (TF001) was deposited at the National Herbarium, Addis Ababa University, Ethiopia.

### Test organisms and experimental animals

The stocks of *T. congolense* that were originally isolated from a pure natural infection of cattle in Arba Minch, Ethiopia were obtained from the School of Veterinary Medicine, Addis Ababa University. The organisms were maintained by serial passages in mice until required as used and described by different workers [15,16]. Swiss albino mice (25–35 g) of both sexes aged 8–12 weeks, obtained from the breeding colony of the Akililu Lemma Institute of Pathobiology, Addis Ababa University, were used for conducting experiments. Protocols for this experiment were in accordance with the guidelines on care and wellbeing of research animals [17] and were approved by research and ethics committee of the School of Pharmacy, Addis Ababa University.

### Preparation of plant extract

The collected plant material was shade dried at room temperature and crushed into powder using laboratory mortar and pestle. The powdered specimen was then extracted by maceration technique using dichloromethane and 80% methanol to obtain the corresponding crude extracts. Briefly, 100 g of the powdered plant material was weighed out and soaked separately in 1000 ml of solvents for 72 hours. The mixture was first filtered using gauze and then the filtrate was passed through sterile filter paper (Whatman No. 3, Whatman Ltd. England). Then the filtered extract was dried in an oven (hydromethanolic extract) at a temperature of 40°C and in rotary evaporator (dichloromethane extract) at 38.5-42°C. The resulting extract was then weighed, recorded and kept in a refrigerator until required for use.

### Acute toxicity study

Acute toxicity study was done using the limit test dose of 2000 mg/kg according to OECD guideline for testing of chemicals using mice [18]. Six female Swiss albino mice separately received orally 2000 mg/kg of the hydromethanolic and DCM extracts. The mice were observed continuously for 1 h after administration of the extracts; intermittently for 4 h, over a period of 24 h and for 14 days for gross behavioral changes and other signs of toxicity manifestations. The acute toxicity study indicated that there were no visible signs of acute toxicity and death was not observed at the limit dose tested (2000 mg/kg) during the 14 days observation period.

### Parasite inoculation and extract administration

The isolates were first inoculated heavily (10^4^ trypanosomes/mouse) to donor mice. After establishment of infection (11–12 days post-inoculation), the donor mice were then subjected to cardiac puncture and blood was collected and immediately diluted with PBS for subsequent *in vivo* test. A total of sixty healthy mice were randomly grouped (n = 6) into ten groups (A-I, A-II, A-III, B-I, B-II, B-III, C, D, E and F). All experimental groups of mice (except group F) were then infected intraperitoneally with 2000 trypanosomes/mouse in 0.2 ml of blood as described and used by Ene *et al*. [19]. The animals were left to develop parasitaemia, and when average parasitaemia became approximately 10^7.03^ parasites/ml, groups A-I, A-II and A-III were administered 100, 200 and 400 mg/kg of the hydromethanolic extract respectively. Whereas groups B-I, B-II and B-III received respectively 100, 200 and 400 mg/kg of the DCM extract. Dose of extracts were selected based on the results of acute toxicity. The extracts were administered to these groups intraperitonneally every morning for seven days. The extracts were dissolved and reconstituted in 2% Tween-80 in distilled water. Group C and D received 3.5 and 28 mg/kg (intraperitoneal single dose) of standard drug, diminazene aceturate, respectively. Group E consisted of the negative control which were infected with the parasite and received the vehicle. The group F included uninfected-untreated mice for reference.

### Determination of parasitaemia and body weight change

The degree of parasitaemia was determined using the method of Herbert and Lumsden [20]. Briefly, the method involves microscopic counting of parasites per field in blood smears from the peripheral blood obtained from tail vein of of each mouse. Wet smears were prepared in triplicates from each animal and the mean value of slide counts was taken per sample examined microscopically. Logarithm values of these counts were obtained by matching with the table given by Herbert and Lumsden [20]. Parasitaemia was monitored every other day until the 14^th^ day and twice a week thereafter.

For the assessment of antitrypanosomal effect of the extracts, the level of parasitaemia (expressed as log of absolute number of parasites per ml of blood) in the treated animals was compared to that of the control animals. The body weight of each mouse in all groups was also measured on the day treatment commenced (day 0) and every other day up to day 14 by a sensitive digital weighing balance.

### Determination of packed cell volume and differential white blood cell count

The peripheral blood obtained from tail vein of of each mouse was used for determination of packed cell volume and differential white blood cell count. PCV was measured on days 0, 7 and 14 using Wintrobe’s method to predict the effectiveness of the test extracts in preventing haemolysis resulting from increasing parasitaemia associated with trypanosomiasis. The differential white blood cell (WBC) counts were determined on Giemsa-stained thin blood films obtained from each mouse on the 14^th^ day of extract administration.

### Determination of mean survival time

Mortality was monitored daily and the number of days from the time of inoculation of the parasite up to death was recorded for each mouse in the treatment and control groups throughout the follow up period for six weeks.

### Statistical analysis

Values of the data obtained from the study were summarized and expressed as mean ± standard error of mean. Data analysis was performed using Statistical Packagefor Social Science (SPSS), version 16.0. To compare the results obtained from different groups, one way ANOVA followed by Tukey’s multiple comparison tests was performed to determine statistical significance. P values less than 0.05 were considered significant.

## Results

### Effect of crude extracts on parasitaemia

There were fluctuations in the level of parasitaemia of all the treated groups which were, however, kept at relatively low or very low level relative to infected-untreated control especially at higher doses of both extracts. The best results were obtained with DCM extract at an optimum dose of 400 mg/kg (Table 1) which was less effective compared with 28 mg/kg of standard drug. Comparison with the negative control revealed that the 400 mg/kg DCM extract has kept parasitaemia at a significantly low level (p < 0.05 on days 4 through 14) and 200 mg/kg had considerably reduced parasitaemia (p < 0.05) on days 6 and 8 of treatment. The mice treated with 400 mg/kg hydromethanolic crude extract had significantly reduced parasitaemia (p < 0.05 on days 6, 8 and 10 of treatment) when compared with the infected-untreated group. Maximum dose (28 mg/kg) of the standard (DA) was able to temporarily clear the parasites from circulation within two days of treatment although parasites reappeared from day 12 of treatment until all animals relapsed on day 16.83 ± 1.28 (Tables 1 and 2). The 3.5 mg/kg DA was not able to even briefly clear the trypanosomes from all animals right from the beginning and relapse in this group was recorded in all animals after day 6.16 ± 1.42 of treatment. Comparison between the groups treated with the two types of extracts indicated that the DCM extract appeared to be superior to the hydromethanolic extract in reducing the parasite burden. The results showed that mice treated with 400 mg/kg of DCM extract had continually reduced parasitaemia level from day 2 of treatment to day 8 which was further kept on average at lowest level up to the end of the monitoring period. However, parasitaemia reduction at higher doses, 400 and 200 mg/kg, of DCM extract and 400 mg/kg hydromethanolic extract were not significantly different.

**Table 1:** **Effect of dichloromethane crude extract of aerial parts of *A. abyssinica *** **on parasitaemia and mean survival time of *T. congolense *** **infected mice**

**Treatment**	**Dose (mg/kg)**	**Parasitaemia level (log number)/ml**								**Mean survival time (days)**
		**D0**	**D2**	**D4**	**D6**	**D8**	**D10**	**D12**	**D14**	
DCM ext	100	6.68 ± 0.23	7.08 ± 0.25	7.57 ± 0.26^bc^	7.83 ± 0.14^bcd^	6.31 ± 0.46^cd^	6.70 ± 1.37^c^	7.31 ± 0.44^cd^	6.41 ± 1.34^c^	34.33 ± 4.63
DCM ext	200	7.05 ± 0.18	7.04 ± 0.23	5.65 ± 1.18^bc^	3.80 ± 1.20^a^	3.92 ± 1.37^a^	6.70 ± 0.30^c^	6.78 ± 0.39^c^	7.07 ± 0.32^c^	37.83 ± 3.49
DCM ext	400	7.00 ± 0.30	6.95 ± 0.26	4.90 ± 0.98^ac^	2.80 ± 1.26^a^	1.95 ± 1.24^a^	3.70 ± 1.17^a^	4.85 ± 0.99^abc^	4.95 ± 1.01^a^	40.67 ± 2.42
DA	3.5	7.09 ± 0.06	1.25 ± 1.25	1.90 ± 1.20^a^	3.30 ± 1.48^a^	4.60 ± 1.48^c^	6.12 ± 1.26^c^	7.41 ± 0.40^cd^	7.92 ± 0.27^c^	36.83 ± 4.36
DA	28	7.13 ± 0.05	0.00 ± 0.00	0.00 ± 0.00^a^	0.00 ± 0.00^a^	0.00 ± 0.00^ab^	0.00 ± 0.00^f^	0.90 ± 0.90^e^	1.85 ± 1.17^f^	42.00 ± 0.00
Distilled water	1 ml	6.94 ± 0.26	7.97 ± 0.12	8.67 ± 0.10	8.54 ± 0.07	8.46 ± 0.17	8.70 ± 0.06	8.14 ± 0.27	8.45 ± 0.21	34.33 ± 6.15

Values are mean ± SEM; n = 6; D = day; D0 = the day treatment commenced; SEM = standard error of mean; DCM = Dichloromethane; ext = extract; DA = Diminazene aceturate; All superscripts indicate significance at p < 0.05 (^a^ = compared to negative control; ^b^ = compared to 3.5 mg/kg DA; ^c^ = compared to 28 mg/kg DA; ^d^ = compared to 400 mg/kg; ^e^ = compared to all groups, ^f^ = compared to all groups but 400 mg/kg).

**Table 2:** **Effect of hydromethanolic crude extract of aerial parts of *A. abyssinica *** **on parasitaemia and mean survival time of *T. congolense *** **infected mice**

**Treatment**	**Dose (mg/kg)**	**Parasitaemia level (log number)/ml**								**Mean survival time (days)**
		**D0**	**D2**	**D4**	**D6**	**D8**	**D10**	**D12**	**D14**	
80% MeOH ext	100	7.16 ± 0.18	8.10 ± 0.17	7.80 ± 0.28^bc^	7.92 ± 0.26^bcd^	7.88 ± 0.16^c^	8.23 ± 0.21^c^	7.82 ± 0.16^c^	8.45 ± 0.13^c^	35.17 ± 6.49
80% MeOH ext	200	7.02 ± 0.09	7.90 ± 0.08	7.57 ± 0.24^bc^	6.90 ± 0.49^c^	6.70 ± 0.47^c^	7.95 ± 0.24^c^	7.69 ± 0.33^c^	8.10 ± 0.27^c^	36.50 ± 5.09
80% MeOH ext	400	7.17 ± 0.32	7.83 ± 0.28	6.05 ± 0.36^bc^	3.00 ± 1.36^a^	3.87 ± 1.37^a^	4.90 ± 1.00^ac^	7.30 ± 0.12^c^	7.15 ± 0.31^c^	38.17 ± 4.88
DA	3.5	7.09 ± 0.06	1.25 ± 1.25	1.90 ± 1.20^ad^	3.30 ± 1.48^a^	4.60 ± 1.48^c^	6.12 ± 1.26^c^	7.41 ± 0.40^c^	7.92 ± 0.27^c^	36.83 ± 4.36
DA	28	7.13 ± 0.05	0.00 ± 0.00	0.00 ± 0.00^ad^	0.00 ± 0.00^a^	0.00 ± 0.00^ab^	0.00 ± 0.00^e^	0.90 ± 0.90^e^	1.85 ± 1.17^e^	42.00 ± 0.00
Distilled water	1 ml	6.94 ± 0.26	7.97 ± 0.12	8.67 ± 0.10	8.54 ± 0.07	8.46 ± 0.17	8.70 ± 0.06	8.14 ± 0.27	8.45 ± 0.21	34.33 ± 6.15

Values are mean ± SEM; n = 6; D = day; D0 = the day treatment commenced; SEM = standard error of mean; MeOH = Methanol; ext = extract; DA = Diminazene aceturate; All superscripts indicate significance at p < 0.05 (^a^ = compared to negative control; ^b^ = compared to 3.5 mg/kg DA; ^c^ = compared to 28 mg/kg DA; ^d^ = compared to 400 mg/kg; ^e^ = compared to all groups).

### Effect of crude extracts on body weight

**Figure 1:**
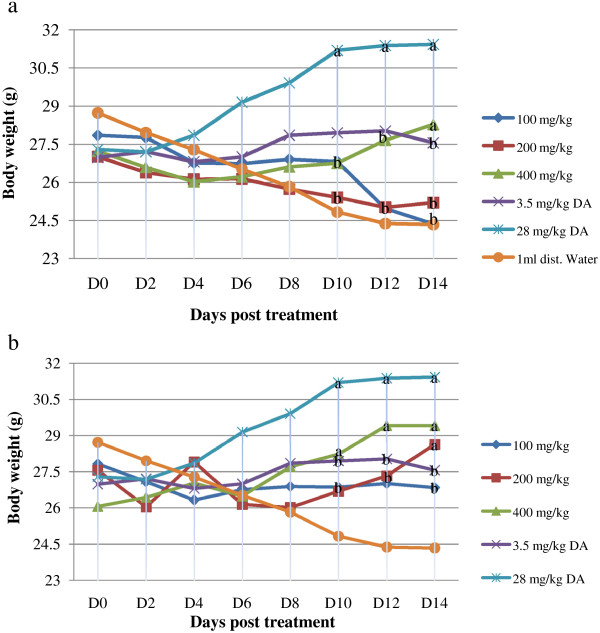
**Effect of extract of *Artemisia abyssinica *on body weight of mice. (a)** hydromethanolic crude extract **(b)** dichloromethane crude extract. Values are mean ± SEM; n = 6; D = day; D0 = the day treatment commenced; a = p < 0.05 compared to negative control; b = p < 0.05 compared to 28 mg/kg DA.

Treatment with the crude extracts prevented loss of weight associated with parasitaemia particularly at 200 and 400 mg/kg dose levels of DCM extract and with 400 mg/kg of hydromethanolic extract compared to the negative controls. There were no detectable differences in preventing weight loss associated with parasitaemia between the extracts as well as between the extracts and standard (3.5 mg/kg) at these dose levels throughout the monitoring period (Figure 1a and b). Considerable body weight improvement was seen by 28 mg/kg DA (12.9%) followed by 400 mg/kg DCM extract (11.42%) relative to the pre-treatment value.

### Effect of crude extracts on packed cell volume

**Figure 2:**
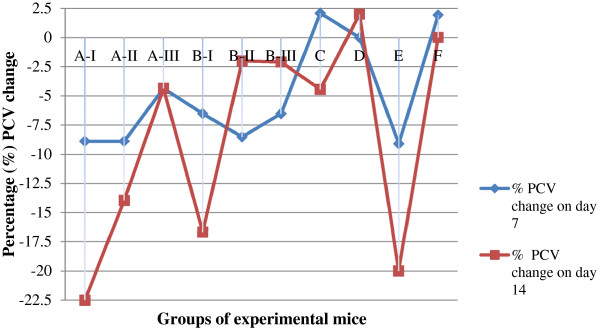
**Percentage packed cell volume change of *T. congolense *infected mice treated with hydromethanolic and dichloromethane crude extracts of aerial parts of *A. abyssinica.*** Values are mean ± SEM; n = 6; PCV = packed cell volume; A = Hydromethanolic extract; B = Dichloromethane extract; I = 100 mg/kg; II = 200 mg/kg; III = 400 mg/kg; C = 3.5 mg/kg DA; D = 28 mg/kg DA; E = Negative control; F = uninfected-untreated.

As shown in Figure 2, administration of the plant extracts to the infected group produced a substantial difference in PCV when compared with the infected-untreated group. The prevention of PCV reduction observed at 200 mg/kg and 400 mg/kg DCM extracts over the infected-untreated group was comparable. These values were also comparable between these dose levels and to that caused by the standard at 28 mg/kg with no statistically significant difference.

### Effect of crude extracts on differential white blood cell count

**Figure 3:**
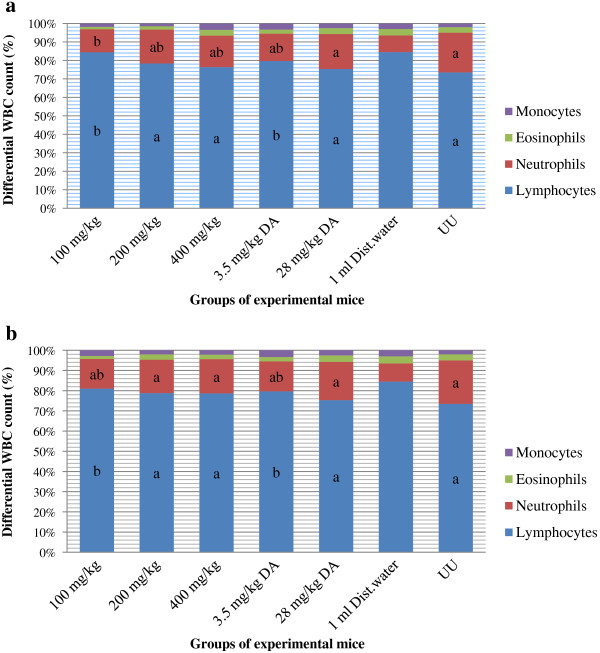
**Effect of extract of *Artemisia abyssinica *on differential white blood cell count of mice. (a)** hydromethanolic crude extract **(b)** dichloromethane crude extract. Values are mean ± SEM; n = 6; UU = uninfected-untreated; WBC = white blood cell; a = p < 0.05 compared to negative control; b = p < 0.05 compared to uninfected-untreated.

The results in Figure 3a and b indicate that differential white blood cell (WBC) count revealed that% lymphocyte counts were significantly higher (p < 0.05) in the groups that received lowest dose (100 mg/kg) of both extracts, 3.5 mg/kg DA and in the infected-untreated group compared to uninfected-untreated group; whereas marked decrease (p < 0.05) in neutrophil count was noticed in these groups.

The lymphocyte and neutrophil levels in the remaining experimental groups were comparable to that in the uninfected-untreated group. Comparison with the negative control, neutrophil levels in all treated groups except 100 mg/kg hydromethanolic extract, showed noticeable increase (p < 0.05) while lymphocyte levels showed marked decrease (p < 0.05) except in those groups that received lowest dose (100 mg/kg) of both extracts and 3.5 mg/kg DA in which the values were similar.

### Effect of crude extracts on mean survival time

Analysis of the mean survival time (days) revealed that only all the mice treated with DA (28 mg/kg) survived throughout the period of the study (42.00 ± 0.00) compared to groups that received the extracts, standard dose of DA (3.5 mg/kg) and the infected-untreated control. Among the extract treated animals, the group treated with DCM extract at 400 mg/kg had the highest mean survival time of 40.67 ± 1.00 days (Tables 1 and 2).

## Discussion

The objective of the present study was to evaluate the *in vivo* antitrypanosomal activity of crude methanolic and dichloromethane extracts of aerial parts of *A. abyssinica* against field isolate of *T. congolense* in mice models of infection. The *in vivo* mice study revealed that the extracts did not completely eliminate the trypanosomes from the blood stream of infected mice, but considerably reduced level of parasitaemia. Though parasitaemia was not completely eliminated, taken as a whole, the DCM extract had the highest antitrypanosomal activity compared to the hydromethanolic extract and the untreated control. Several other workers also made similar observation on the differentially high antitrypanosomal activity produced by lipophilic extracts (DCM, chloroform, petroleum ether) of different plants both *in vivo* and *in vitro* (10, 15,19). The finding is in agreement with our former report which showed that DCM extract had better *in vitro* antitrypanosomal activity than hydromethanolic extract [14].

The hydromethanolic extract similarly reduced parasitaemia but only at its optimum dose of 400 mg/kg body weight. The high parasite load attained before commencement of treatment, enzymatic inactivation of active compounds and impaired absorption from the site of administration could mask efficacy of crude extracts to completely clear the parasites in the blood [21-23]. The fact that the plant showed differential activity between extracts is confirmation of earlier assertions [24] that any statement on a plant’s trypanocidal activity should be taken within the context of the plant part and the solvent extract tested. This is because difference in solvent of extraction may reveal different phytochemicals in the same plant. A similar finding to earlier reports [15,19,24] in the difference in composition of secondary metabolites was observed with the two solvents of extraction in this investigation which could in part contribute to the activity difference.

Although diminazene aceturate was able to temporarily clear trypanosomes from circulation of infected mice, relapse occurred in all experimental mice approximately on days 6 and 16 of treatment respectively at 3.5 and 28 mg/kg dose levels. This observation was not surprising as trypanocidal drug resistance has been wide spread in several African countries including Ethiopia [25-28].

The present study did not involve detailed characterization and isolation of different compounds that could be responsible for the observed activity. However, phytochemical screening revealed the presence of tannins, flavonoids, alkaloids, cardiac glycosides and polyphenols in the hydromethanolic extract, and terpenoids, polyphenols and phytosteroids in the DCM extract [14].

The antitrypanosomal effect shown by extracts in this study might be attributed to the presence of one or more of these constituents which, in contrast to synthetic pharmaceuticals based upon single chemicals, may exert their effects through the additive or synergistic action of several chemical compounds acting at a single or multiple target sites associated with a physiological process [29].

Several possible mechanisms working separately or in concert may account for the observed effect [30]. Accumulated evidences suggest that many natural products exhibit their antitrypanosomal activity by virtue of their interference with the redox balance of the parasites acting either on the respiratory chain or on the cellular defenses against oxidative stress [24]. This is because natural products possess structures capable of generating radicals that may cause peroxidative damage to trypanothione reductase that is very sensitive to alterations in redox balance. Published reports also indicate that several natural products such as alkaloids, polyphenols, terpenoids and saponins that primarily interact with important molecular targets such as DNA, microtubules, biomembranes, receptors and may induce cytotoxicity and death in trypanosomes. Several active alkaloids are known to intercalate DNA, and in consequence inhibit DNA and RNA polymerase, topoisomerases, ribosomal protein biosynthesis, or bind to tubulin/microtubules, thus acting as spindle poisons to disturb membrane integrity in trypanosomes [31].

Trypanosome generated reactive oxygen species can also attack red blood cells’ membranes, induce oxidation and subsequently hemolysis. Thus, scavenging the trypanosome associated free radicals by phytochemicals such as flavonoids and other polyphenolic antioxidants may ameliorate anemia induced by trypanosome infection [32]. The plant extracts may strengthen the host defense that was already activated because of the presence of trypanosomes in circulation with established infection [33].

The mechanism by which these extracts exhibited their antitrypanosomal activity can only be speculated since the active ingredient(s) were not isolated. Several investigations tend to suggest that it is often difficult to speculate and decipher the exact mode of action by which plant extracts exhibit their trypanocidal action. Indeed, the possible mechanisms by which plant extracts and phytochemicals therein carry out this role remain a subject of great speculations and debate in the scientific community [34]. Thus, for the DCM extract in our study, the antitrypanosomal effect produced would most likely be ascribed to either of the terpenoids, polyphenols or phytosteroids detected in the extract. However, rare findings have suggested as to how any of these phytochemicals produce their antitrypanosomal activity. The result of Nibret *et al.*[10] has clearly indicated that out of the 40 plant extracts tested, the DCM extract from stem bark of *Warburgia salutaris* (claimed to be used against many pathologies in many parts of Africa) was found to exhibit the most potent trypanocidal activity. The trypanocidal activity was suggested to be due to the drimane sesquiterpenoids (warburganal and polygodial). Concerning the mechanism, it was proposed that the two sesquiterpene aldehydes, warburganal and polygodial, formed covalent bonds with amino groups of proteins and affect a vast number of cellular activities.

The positive effect of the extracts against trypanosome infection can further be deduced from the weight status of the animals and the body weight improvement was consistent with the observation made on parasitaemia. This observation indicates that the animals with better physical state could feed more than those in the other groups and resist weight loss that is usually associated with trypanosomiasis.

The observed antitrypanosomal effect of the extracts in this study was accompanied by corresponding improvement and prevention of further drop in PCV suggesting that they have potentials to ameliorate anaemia. This could possibly be by reducing the proliferating parasite load, neutralizing the toxic metabolites produced by trypanosomes or scavenging the trypanosome associated free radicals [15,21,35]. This was particularly noticed in the second week of treatment, in groups with significantly reduced parasitaemia, where the final PCV values were almost similar to the pre-treatment values. The infected mice treated with the standard drug (diminazine aceturate) at 28 mg/kg showed nearly stable PCV up to day 14 of treatment although relapse occurred in all animals after 16 days post treatment. In contrast, the recommended standard dose for cattle and mice (3.5 mg/kg) was unable to prevent PCV reduction suggesting its failure to eliminate parasitaemia. Similar observations have been reported in previous studies [26,36,37].

Lymphocytes are the main effector cells of the immune system [38]. Results of the differential white blood cell count revealed that higher differential lymphocyte counts were generally recorded in those groups in which parasitaemia was not relatively kept in check and in the infected-untreated. Leucocytosis which may be due to lymphocytosis have been implicated in trypanosomiasis and these conditions are usually as a result of wax and wear syndrome on the animal immune system caused by the ever changing variable surface glycoprotein of the infecting trypanosomes [39] that demands the immune system to continuously produce antibodies and hence keep the level of lymphocytes high. However, administration of the extracts that have significantly lowered parasitaemia seemed to prevent lymphocytosis suggesting that this phenomenon is a result of parasite load. Hence, the relatively reduced percentage of lymphocytes post extract treatment suggests that the plant extracts by suppressing parasitaemia contributed to the attenuation of the immunological reaction.

The lower differential counts of neutrophils observed in the infected negative control and groups in which parasitaemia was persistently higher may be attributed to the immunosuppressive actions of trypanosome infection [39-41]. Several mechanisms such as granulocyte hypoplasia and splenic sequestration have been proposed for neutropenia in African trypanosomiasis [42]. It has been suggested that granulocyte progenitor cells may be coated with trypanosome antigen-antibody as occurs with RBC leading to phagocytosis [43]. In this study, we suggest that treatment with crude extracts of aerial parts *A. abyssinica* has reduced parasite load consequently leading to a decrease in coating of the progenitor cells which prevented or reduced the development of neutropenia.

Neither the extracts nor the standard drug cured the infection although parasitaemia was kept in check. Earlier reports indicated that in *T. congolense* infections probably the long lasting chronic wasting situation and a complex process of gradual impairment of organs and tissues of the host lead to the eventual generalized collapse. A sufficiently balanced situation in the host-parasite relationship might allow the establishment of a chronic infection that could lead to survival of infected animals for several months [44]. The mice in all groups that showed high levels of parasitaemia survived for at least 34 days showing that they resisted the parasites for appreciable period of time.

## Conclusion

*In vivo* assay of the present investigation has provided evidence that extracts of aerial parts of *A. abyssinica* have reduced parasite burden and ameliorated anaemia, prevented body weight loss, increased neutrophil levels and decreased lymphocyte levels as a consequence of the parasites being kept in check and DCM extract showed a superior antitrypanosomal activity than hydromethanolic extract. This study established that aerial parts of *A. abyssinica* have antitrypanosomal potential and can be considered a potential source of new drugs for the treatment of tropical diseases caused by trypanosomes.

## Abbreviations

DCM: Dichloromethane; DA: Diminazine aceturate; T.congolense: *Trypanosoma concolense*; A. abyssinica: *Artemisia abyssinica*; PCV: Packed cell volume; WBC: White blood cell.

## Competing interests

The authors declare that they have no competing interests.

## Authors’ contributions

TF conceived the study, designed and conducted all laboratory experiments; analysed and interpreted experimental results. WS and GT participated in the proposal, study design and manuscript preparations. All authors read and approved the final manuscript.

## Pre-publication history

The pre-publication history for this paper can be accessed here:

http://www.biomedcentral.com/1472-6882/14/117/prepub

## References

[B1] Brun R Hecker H Lun ZR *Trypanosoma evansi* and *T. equiperdum*: distribution, biology, treatment and phylogenetic relationship (a review) Vet Parasitol 1998 79 95 107 10.1016/S0304-4017(98)00146-0 9806490

[B2] Swallow BM Impacts of trypanosomiasis on African agriculture Rome: PAAT Technical and Scientific Series 2000 2 52

[B3] Patrick M Epco H Veerle L Victor K Jean-Jacques M Pascal L Marleen B Human african trypanosomiasis diagnosis in first-line health services of endemic countries, a systematic review PLoS Negl Trop Dis 2012 6 11 e1919 10.1371/journal.pntd.0001919 23209860 PMC3510092

[B4] John WH Rachid O Damian K Glyn AV Stephen JT Modeling the control of trypanosomiasis using trypanocides or insecticide-treated livestock PLoS Negl Trop Dis 2012 6 5 e1615 10.1371/journal.pntd.0001615 22616017 PMC3352824

[B5] Geerts S Holmes PH Drug management and parasite resistance in animal trypanosomiasis in Africa.Position Paper-ProgrammeAgainst African Trypanosomiasis (PAAT) Technical series 1998 Rome, Italy: FAO 22

[B6] Delespaux V Geysen D Van den Bossche P Geerts S Molecular tools for the rapid detection of drug resistance in animal trypanosomes Trends Parasitol 2008 24 236 242 10.1016/j.pt.2008.02.006 18420457

[B7] Legros D Ollivier G Gastellu-Etchegorry M Paquet C Burri C Jannin J Buscher P Treatment of human Africantrypanosomiasis-present situation and needs for researchand development Lancet Infect Dis 2002 2 437 440 10.1016/S1473-3099(02)00321-3 12127356

[B8] Hoet S Opperdoes F Brun R Adjakidje V Quetin L Eclercq J In vitro antitrypanosomal activity of ethnopharmacologically selected Beninese plants J Ethnopharmacol 2004 91 37 42 10.1016/j.jep.2003.11.008 15036465

[B9] Nibret E Wink M Volatile components of four Ethiopian Artemisia species extracts and their in vitro antitrypanosomal and cytotoxic activities Phytomedicine 2010 17 369 374 10.1016/j.phymed.2009.07.016 19683909

[B10] Nibret E Ashour ML Rubanza CD Wink M Screening of some Tanzanian medicinal plants for their trypanocidal and cytotoxic activities Phytother Res 2010 24 945 947 19957246 10.1002/ptr.3066

[B11] Yineger H Kelbessa E Bekele T Lulekal E Ethnoveterinary medicinal plants at Bale Mountains National Park, Ethiopia J Ethnopharmacol 2007 112 55 70 10.1016/j.jep.2007.02.001 17368989

[B12] Geyid A Abebe D Debella A Makonnen Z Aberra F Teka F Kebede T Urga K Yersaw K Biza T Haile Mariam B Guta M Screening of some medicinal plants of Ethiopia for their anti-microbial properties and chemicals profiles J Ethnopharmacol 2005 97 421 427 10.1016/j.jep.2004.08.021 15740876

[B13] Tadesse M Hedberg I, Friis IB, Edwards S Asteraceae (Compositae) Flora of Ethiopia and Eritrea, Volume 4, part 2 2004 Uppsala University, Sweden: The National Herbarium, Biology Department Science Faculty, Addis Ababa University, Ethiopia, and The Department of Systematic Botany 222 223

[B14] Feyera T Terefe G Shibeshi W Phytochemical screening and in vitro antitrypanosomal activity of the aerial parts of *Artemisia abyssinica* against Trypanosoma congolense field isolate Ethiopian Pharmaceutical Journal 2011 29 137 142

[B15] Ogoti P Esther M Joanna A Gabriel M Mabel I Grace M Evaluation of *in vivo* antitrypanosomal activity of selected medicinal plant extracts J Med Plant Res 2009 2 849 854

[B16] Maikai VA Antitrypanosomal activity of flavonoid extracted from *Ximenia americana* stem bark Int J Biol 2011 1 115 121

[B17] Institute for laboratory animal research (ILAR) Guide for the care and use of laboratory animals 1996 Washington, D.C: National Academy Press

[B18] Organization of Economic Co-operation and Development The OECD guidelines for testing of chemical: 423 acute oral toxicity 2001 France: OECD Publishing

[B19] Ene AC Atawodi SE Ameh DA Nnamani CN Apeh YEO Antitrypanosomal effects of petroliume ether, chloroform and methanol extracts of *Artemisia maciverae* Linn Indian exp Biol 2009 47 981 986 20329702

[B20] Herbert WJ Lumsden WHR Trypanosoma brucei. a rapid matching method for estimating the host’s parasitaemia Expt Parasitol 1976 40 427 431 10.1016/0014-4894(76)90110-7 976425

[B21] Ekanem JT Kolawole OM Abbah OC Trypanocidal potential of methanolic extract of *Bridelia ferruginea* benth bark in *Rattus novergicus* Afr J Biotechol 2008 2 45 50

[B22] Antia RE Olayemi JO Aina OO Ajaiyeoba EO *In vitro* and *in vivo* animal model antitrypanosomal evaluation of ten medicinal plant extracts from southwest Nigeria Afr J Biotechol 2009 8 1437 1440

[B23] Mann A Egwim EC Banji B Abdukadir N Gbate M Ekanem JT Efficacy of *Dissotisro tundifolia* on *Trypanosoma brucei brucei* infection in rats Afr J Biochem Res 2009 3 5 8

[B24] Atawodi SE Bulus T Ibrahim S Ameh DA Nok AJ Mamman M Galadima M In vitro trypanocidal effect of methanolic extract of some Nigerian savannah plants Afr J Biotechol 2003 2 317 321

[B25] Afewerk Y Clausen PH Abebe G Tilahun G Mehlitz D Multiple-drug resistant *T. congolense* population in village cattle of Metekel district, northwest Ethiopia Acta Trop 2000 76 231 238 10.1016/S0001-706X(00)00108-X 10974163

[B26] Chaka H Abebe G Drug resistant trypanosomes: a threat to cattle production in the Southwest of Ethiopia Revue d’Elevageet de MédecineVétérinaire des Pays Tropicaux 2003 56 33 36

[B27] Adamu M Nwosu CO Igbokwe IO Toxicity and phytochemical constituents of aqueous extract of *Ocimum gratissimum* leaf Nig Vet J 2008 29 48 57

[B28] Miruk A Hagos A Yacob HT Asnake F Basu AK Prevalence of bovine trypanosomiasis and trypanocidal drug sensitivity studies on *T. congolense* in Wolyta and Dawero zones of southern Ethiopia Veterinary Parasitol 2008 152 141 147 10.1016/j.vetpar.2007.12.007 18207329

[B29] Tyler VE Phytomedicines: back to the future J Nat Prod 1999 62 1589 1592 10.1021/np9904049 10579884

[B30] Paul F Etet S Mahomoodally FM New insights in staging and chemotherapy of African trypanosomiasis and possible contribution of medicinal plants Sci World J 2012 343652 1 16 10.1100/2012/343652 PMC3349134 22593674

[B31] Rosenkranz V Wink M Alkaloids induce programmed cell death in blood stream forms of Trypanosomes (*Trypanosoma b. brucei*) Molecules 2008 13 2463 2473 10.3390/molecules13102462 PMC6244846 18833031

[B32] Karori SM Ngure RM Wachira FN Wanyoko JK Mwangi JN Different types of tea products attenuate inflammation induced in *Trypanosoma brucei brucei* infected mice Parasitol Internat 2008 57 325 333 10.1016/j.parint.2008.02.003 18456544

[B33] Okeke N The haematological properties of M .balsamina fruit pulp extract in rabbits 2004 Nigeria: BMLS project, College of Medical Laboratory Sciences, University of Jos

[B34] Gurib-Fakim A Mahomoodally MF African flora as potential sources of medicinal plants: towards the chemotherapy of major parasitic and other infectious diseases- A review JJBS 2013 6 2 77 84 10.12816/0000263

[B35] Mpiana PT Tshibanga DST Shetonde OM Ngbolua KN *In vitro* antidrepanocytary activity (antisickle cell anaemia) of some Congolese plants Phytomedicine 2007 14 192 195 10.1016/j.phymed.2006.05.008 17113273

[B36] Geerts S Delespaux V Van den Bossche P Drug resistance in trypanosomes of livestock: a worrying issue Meded Zitt K Acad Overzeese Wet 2010 55 177 184

[B37] Chitanga S Marcotty T Namangala B Van den Bossche P Van Den Abbeele J High prevalence of drug resistance in animal trypanosomes without a history of drug exposure PLoSNegl Trop Dis 2011 5 1454 10.1371/journal.pntd.0001454 PMC3243716 22206039

[B38] Alli LA Okochi VI Adesoken AA Antitrypanosomal activity and haematological effects of aqueous extract of leaves of Morinda lucida on Trypanosoma brucei brucei infected rats Asian J Pharm Hea Sci 2011 1 111 115

[B39] Abubakar A Iliyasu B Yusuf AB Igweh AC Onyekwelu NA Shamaki BU Afolayan DA Ogbadoyi EO Antitrypanosomal and haematological effects of selected Nigerian medicinal plants in Wistar rats Biokemistri 2005 17 95 99

[B40] Ekanem JT Yusuf OK Some biochemical and haematological effects of black seed (*Nigella sativa*) oil on *Trypanosoma brucei* infected rats Afr J Biotech 2008 7 153 157

[B41] Ezebuiro OGC Yohanna JA Abdulya’aq A Osue HO Abenga JN Yakasai MA Attahir A Haematological changes following acute *trypanosoma brucei brucei* infection in rabbits Curr Res J BiolSci 2012 4 414 416

[B42] Anosa VO Haematological and biochemical changes in human and animal trypanosomiasis part II Revue Elev Med Vet Paystrop 1988 2 151 164 3064194

[B43] Kaaya GP Tizard I Maxie M Valli VEO Inhibition of leucopoiesis by sera from *T. congolense* Tropenmed Parasit 1980 31 232 238 7414680

[B44] Takeya M Reinwald E Risse HJ Pathobiochemical alterations in experimental chronic and acute trypanosomal infection in mice J Clin Chem Clin Biochem 1987 25 665 673 3694125 10.1515/cclm.1987.25.10.665

